# Whole genome surveying and metabolomic profiling aided navigation of therapeutic plethora of *Streptomyces melanogenes* WPF1 isolated from *Pinus patula* rhizosphere

**DOI:** 10.1186/s40643-025-00973-7

**Published:** 2025-12-10

**Authors:** Abhirami Chithrakumari Ranesan, Vipin Mohan Dan, Abhijeeth Muralidharan Nair, Remya Pattaruparambil, Athira Santhosh, Sajith Raghunandanan, Sarika Ambika Rajendran

**Affiliations:** 1 Division of Microbiology, KSCSTE- Jawaharlal Nehru Tropical Botanic Garden and Research Institute, Pacha-Palode, Thiruvananthapuram, Kerala 695562 India; 2 Research Center, University of Kerala, Thiruvananthapuram, Kerala 695 014, India; 3 Rajiv Gandhi Centre for Biotechnology (RGCB), Cancer Research Programme-4, Thycaud Post, Poojappura, Thiruvananthapuram, Kerala 695 014 India; 4 Department of Public Health and Genomics, Manipal School of Life Sciences, Manipal Academy of Higher Education, Manipal, Karnataka 576104 India; 5 Kerala Biotechnology Commission, KSCSTE, Sasthra Bhavan, Pattom, Thiruvananthapuram, Kerala 695004 India

**Keywords:** *Streptomyces melanogenes*, Antimicrobial, Anticancer, Kinamycin D, antiSMASH, GC–MS, LC–MS

## Abstract

Rhizosphere microbial diversity from Western Ghats remains a relatively less explored source of therapeutic agents. Targeted screening of actinomycetes from an under studied area led to the isolation of 24 actinomycetes from *Pinus patula* rhizosphere. Screening led to identification of *Streptomyces melanogenes* WPF1 with strong therapeutic potential. Delineation of the whole genome of WPF1 and metabolic profiling of the partially purified active fraction (PF10) led to confirmation of kinamycin D, oleic acid, palmitic acid and other components. These molecules in unison can be correlated to the recorded biological activities of the active fraction. AntiSMASH analysis of the WPF1 genome identified 26 biosynthetic gene clusters (BGCs) associated with secondary metabolite biosynthesis, including one cluster exhibiting over 97% similarity to known Kinamycin biosynthetic pathway. LC–MS and high-resolution mass spectrometry (HRMS) analyses revealed presence of Kinamycin D in the partially purified fraction PF10. PF10 demonstrated selective antibacterial activity against Gram-positive human pathogens and effectively inhibited their biofilm formation. Additionally, PF10 suppressed the proliferation of cancer cell lines, with IC_50_ values ranging from 2.0 to 2.5 µg/mL, and induced apoptosis, as evidenced by Poly (ADP-ribose) polymerase (PARP) cleavage, activation of caspase 3 and caspase 9 and Bax protein accumulation. These findings highlight *S. melanogenes* WPF1 as a promising source of bioactive secondary metabolites, and importance of rhizosphere microbial diversity for potential therapeutic discovery.

**Graphical abstract:**

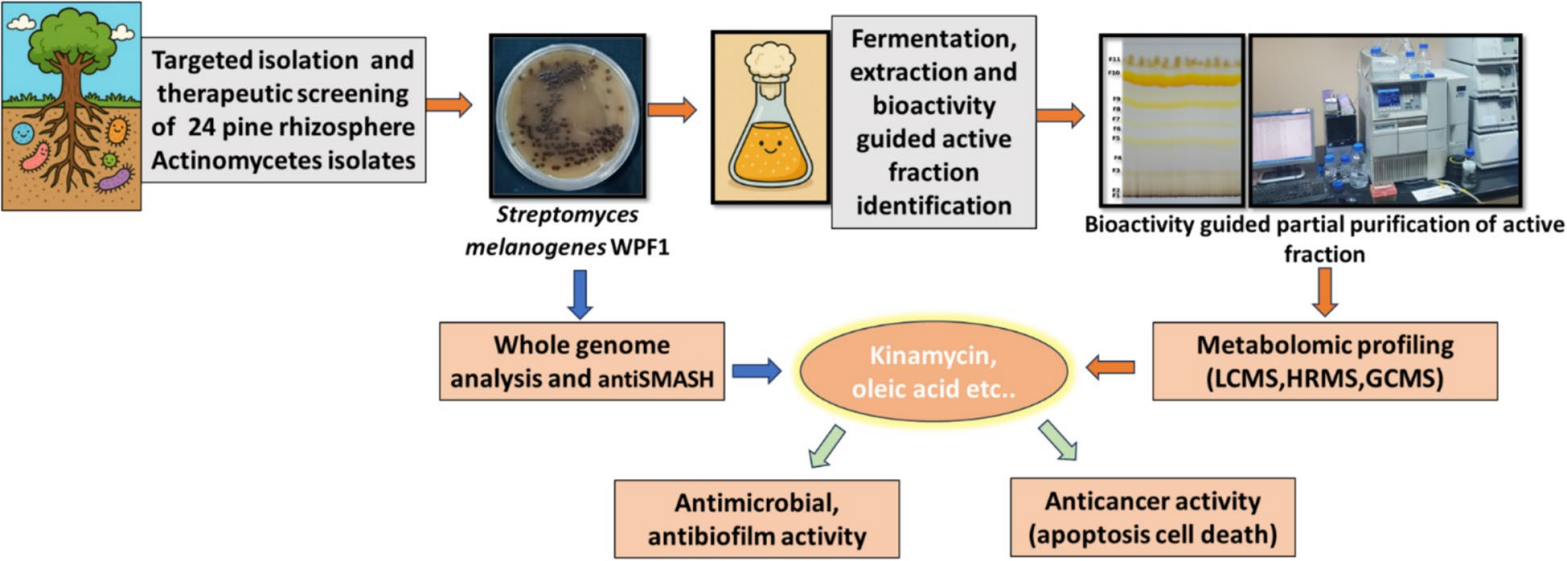

**Supplementary Information:**

The online version contains supplementary material available at 10.1186/s40643-025-00973-7.

## Introduction

The escalating prevalence of antibiotic-resistant pathogens and drug-resistant malignancies underscores the urgent demand for novel antimicrobial and anticancer therapeutics (Harald 2024). According to the 2024 World Health Organization (WHO) priority list, *Staphylococcus aureus*, *Neisseria gonorrhoeae*, and rifampicin-resistant *Mycobacterium tuberculosis* are classified as critical threats, reinforcing the necessity for innovative drug discovery approaches and strengthened global strategies to combat antimicrobial resistance (AMR) (Hatim et al. 2025). Concurrently, cancer remains a leading cause of mortality, accounting for nearly 10 million deaths annually, thereby necessitating continuous progress in diagnostic technologies, therapeutic monitoring, and the development of effective treatment modalities to mitigate the global cancer burden (Miranda et al. 2018).

Natural products, particularly those derived from microorganisms; represent a vital reservoir of structurally diverse bioactive compounds with broad therapeutic potential (Challinor and Bode 2015). Among microbial sources, actinomycetes—most notably members of the genus *Streptomyces*—are recognized as prolific producers of secondary metabolites with remarkable pharmacological relevance (Harir et al. 2018; Muralikrishnan et al. 2017). It is estimated that nearly 45% of all known bioactive microbial metabolites are attributed to actinomycetes, with *Streptomyces* species alone accounting for more than 80% of clinically employed antibiotics, including cornerstone agents such as streptomycin, tetracycline, erythromycin, and the anticancer compound doxorubicin (Demain and Sanchez 2009; Siddharth et al. 2020). This species is widely distributed across terrestrial, marine, and extreme ecosystems, where their adaptive biosynthetic capacities give rise to chemically diverse secondary metabolites. Many of these molecules exhibit potent antibacterial, antifungal, antiviral, anticancer, and immunomodulatory activities, highlighting their indispensable role in the discovery and development of novel therapeutics to address pressing global health challenges (Khorshed et al. 2022; Dan et al. 2024).

The Western Ghats of India, recognized as a UNESCO World Heritage biodiversity hotspot, extend parallel to the western coastline and harbour an extraordinary concentration of endemic flora and fauna (Khaleel 2016; Easa and Rajesh 2025). Rhizosphere actinomycetes in ecologically endemic regions may harbour a wealth of unexplored therapeutic potential. Despite the ecological richness of the Western Ghats, microbial diversity—particularly actinomycetes of rhizosphere origin—remains poorly documented. This gap motivated the present study, focusing on rhizosphere actinomycetes. The Vagamon Pine Forest in Kerala represents one such understudied niche, where the isolation of *Streptomyces melanogenes* WPF1 provides a valuable opportunity to investigate the biosynthetic potential of actinomycetes from underexplored environments. Addressing this knowledge gap, the present study focuses on the isolation and characterization of bioactive metabolites from *S. melanogenes* WPF1, with the aim of expanding the repertoire of therapeutically relevant natural products.

Microbial natural product discovery has entered a transformative “deep-mining era,” enabled by advances in high-throughput sequencing, ultra-sensitive high-resolution mass spectrometry (HRMS), and integrated multi-omics approaches. Genome mining tools such as antiSMASH and DeepBGC have revolutionized the systematic identification of cryptic biosynthetic gene clusters (BGCs), while metabolomics platforms, including UPLC-QTOF-MS/MS and GNPS, allow precise structural characterization of metabolites. The integration of genomics and metabolomics thus provides a robust multidimensional framework for correlating biosynthetic potential with metabolite output, substantially accelerating the discovery of novel bioactive scaffolds. Recent identifications of nocathioamides, mandimycin, and somalactams highlight the efficacy of such integrated strategies in revealing structurally unique natural products with therapeutic promise (Zhaochao et al. 2025).

In this study, we explored rhizosphere actinomycetes from the Vagamon Pine Forest in the Western Ghats, leading to the isolation of *Streptomyces melanogenes* WPF1. Integrated genomic and metabolomic analyses identified Kinamycin D and bioactive lipid moieties, with the strain showing strong antibacterial, antibiofilm, and anticancer activities. These findings highlight the therapeutic potential of rhizosphere actinomycetes and demonstrate the power of combining genome mining with metabolomic validation.

## Materials and methods

### Soil sample collection and screening for actinomycete isolates

Rhizosphere soil samples (eight in total) were collected from different locations within the Vagamon Pine Forest in Kottayam District, Western Ghats, Kerala, India (sampling site coordinates: 76°55′32.304″E, 9°39′22.068″N). Soil adhering closely to the roots of pine trees was sampled at a depth of 5–10 cm using sterile spatulas to target rhizosphere soil**.** The collected samples were kept separately (without pooling) in sterile polythene bags and transported to the laboratory under refrigerated conditions. For the initial screening of actinomycetes, serial dilutions of each rhizosphere soil sample were prepared, and 100 µL aliquots from each dilution were spread onto Starch Casein Agar (SCA) plates supplemented with nystatin (50 mg/L) and nalidixic acid (20 mg/L) to selectively inhibit fungal and Gram-negative bacterial growth, respectively. The plates were incubated at 27 ± 2 °C for 8 days.

### Submerged fermentation and preliminary screening

All selected isolates were grown in ISP2 medium with 0.2% calcium carbonate for 7–8 days at 27 ± 2 °C. After 8 days of incubation, cultures were extracted with ethyl acetate at a 1:1 solvent-to-culture ratio; the obtained extract was concentrated, weighed, and stored under –20 °C for further use. The extracts (500 µg) were tested employing disc diffusion assay in biological triplicates against two Gram-positive bacteria, *Staphylococcus aureus* MTCC740 and *Streptococcus pyogenes* ATCC 19615, and two Gram-negative bacteria, *Pseudomonas aeruginosa* MTCC741 and *Escherichia coli* MTCC443. These four pathogens were selected as a representative panel for the following reasons; *S. pyogenes* is one of the most prevalent infectious agents in global scenario and counted among the most reported nosocomial infections (Avire et al. 2021); *S. aureus* is a clinically significant pathogen known for antibiotic resistance and immune evasion (Hatim et al. 2025); *P. aeruginosa* is a notorious opportunistic pathogen with intrinsic resistance mechanisms and has ability to produce multiple virulence factors causing clinical roadblocks in treatment (Letizia et al. 2025) and *E. coli* is a model Gram-negative pathogen (Braz et al. 2020). Also including two Gram-positive and two Gram-negative bacteria provided a balanced evaluation of the antimicrobial spectrum of the isolates.

### Evaluation of bioactivity

The dried ethyl acetate extract of WPF1 was dissolved in DMSO, and varying concentrations (50, 100, 250, and 500 µg) were applied to sterile discs for antimicrobial and antifungal testing using disc assay**.** The list of pathogens tested is presented in Supplementary information SI 1. Bacterial strains were cultured on Mueller–Hinton agar (MHA) and broth (MHB), while fungal strains were grown on Potato Dextrose Agar (PDA) and Broth (PDB). Nystatin served as the positive control for antifungal assays, and ciprofloxacin was used as the positive control for antibacterial assays, with DMSO as the negative control. All plates were incubated at 37 °C for 18–24 h, and the Zones of inhibition (mm) were measured.

### Genomic DNA extraction

The selected microbe was cultured in YEME medium with 30% sucrose and 0.5% glycine for the isolation of high molecular weight genomic DNA for whole genome sequencing. The organism was incubated at 27 ± 2 °C at 180 rpm for three days. Genomic DNA extraction was performed using the Lucigen kit, and its concentration was measured using the Qubit DNA High Sensitivity assay kit (Invitrogen). The quality of the DNA was evaluated through agarose gel electrophoresis.

### Genome sequencing, data pre-processing, and denovo assembly

The genome was sequenced using the Illumina Novaseq 6000 platform with a 150PE protocol. Quality assessment was conducted using FastQC (Andrews 2010) and MultiQC (Ewels et al. 2016), evaluating base call quality, % bases above Q20 and Q30, %GC content, and adapter contamination. Pre-processing involved adapter removal and trimming of low-quality bases using fastp v0.12.4 (Chen et al. 2018). The resulting paired-end reads were subjected to de novo assembly using Unicycler v0.5.0 (Wick et al. 2017), followed by removal of contigs shorter than 200 bp. Assembly statistics were assessed using QUAST (Gurevich et al. 2013), and assembly quality was validated through read mapping using bowtie2 v2.4.5 (Langmead and Salzberg 2012). Genome completeness was evaluated using BUSCO v5.3.2 (Waterhouse et al. 2018) with the bacteria_odb10 reference.

### Annotation and analysis

Contigs were annotated against the NCBI nucleotide database using BLAST v2.13.0 (Altschul et al. 1990) and Prokka v1.13(Seemann 2014), with additional annotation via a BLAST search against the Uniref100 database. The assembled genome was visualized using Proksee (Proksee. xxxx), featuring key genomic information such as BLAST results, GC Content, CDS, and tRNA. KEGG and COG annotations were obtained by submitting the protein sequences to KAAS (KEGG Automatic Annotation Server) (Moriya et al. 2007) and WebMGA (Wu et al. 2011), respectively. The presence of biosynthetic gene clusters (BGCs) in the genome was predicted using the AntiSMASH 5.0 server.

### Phylogenetic and comparative genomic analysis

Phylogenetic and comparative genomic analysis were conducted using the web-based Type (Strain) Genome Server (TYGS) (Meier-Kolthoff and Göker 2019; Meier-Kolthoff et al. 2022). The analysis involved generating a Whole Genome-based phylogenetic tree, calculating the closest genomes using the MASH algorithm, and determining precise distances using the GBDP (Genome BLAST distance phylogeny) approach under the ‘GreedyWithTrimming’ algorithm and distance d5. Phylogenetic tree construction was performed using FASTME and visualized with PhyD3. Taxonomy identification was performed through Ribosomal Multi-locus Sequence Typing (rMLST) (Jolley et al. 2018). For comparative analysis, the genome of WPF1 was compared with 17 publicly available *Streptomyces* genomes, including its closest relative *S. melanogenes* JCM 4398, to assess evolutionary relationships.

### Thin layer chromatography and direct bioautography

The concentrated ethyl acetate extract of *S. melanogenes* WPF1 was subjected to Thin Layer Chromatography (TLC) for metabolite separation. TLC was performed on Silica Gel 60 F254 aluminum plates (Merck) using different combinations of solvents as the mobile phase.

For Direct Bioautography, agar media (Mueller–Hinton Agar for *S. aureus* and Starch Casein Dextrose Agar for *S. pyogenes*) was poured onto Petri dishes and allowed to solidify. The developed TLC plates were then carefully placed onto the solidified agar surface. Soft agar (0.7% agar), mixed with a suspension of the test organisms (*S. aureus* and *S. pyogenes*), was overlaid on the TLC plates and incubated. Zones of inhibition observed after incubation indicated fractions responsible for antimicrobial activity. Bioactive fractions were scraped from the silica gel, extracted with ethyl acetate, concentrated, dried, and weighed.

### Preparative HPLC

Preparative HPLC was performed using a Shimadzu RP-HPLC system equipped with a CBM-20A system controller, SPD-M40A photodiode array detector, LC-20AP solvent delivery pump, and a Shim-pack GIST C18 column (250 mm × 10 mm, 5 µm particle size). A gradient elution method using methanol and water was employed to achieve effective separation**,** with a flow rate of 15.12 mL/min**.** The bioactive fraction from TLC, was dissolved in methanol, filtered through a 0.45 µm membrane, and 1000 µL was injected into the system. Elution was monitored at 254 nm, and fractions were collected manually based on retention times.

## Metabolomic profiling

### GC–MS analysis

Gas chromatography–mass spectrometry (GC–MS) analysis was performed using a Shimadzu Nexis GC-2030 system equipped with an AOC-30/20i auto-sampler and an SH-I-5Sil MS column (30 m × 0.25 mm × 0.25 µm). Compound identification was carried out using the NIST 20 library.

#### LC–MS analysis

LC–MS analysis was conducted using a Vanquish UHPLC system coupled with an Eclipse Orbitrap mass spectrometer. Separation was achieved on a Waters RP column (2.1 × 150 mm, 1.8 µm particle size) using a binary gradient of 0.1% formic acid in water (A) and methanol (B). Data acquisition was performed in both positive and negative ion modes, and compound annotation was aided by mzCloud, ChemSpider, KEGG, and LIPID MAPS databases.

#### HRLC–MS–QTOF

High-resolution LC–MS analysis was performed on an Agilent QTOF system with a Hypersil GOLD C18 column (100 × 2.1 mm, 3 µm) operating in positive ion mode. Data were acquired in AutoMS^2^ mode and processed using Agilent Mass Hunter software (B.06).

Detailed operating parameters for GC–MS, LC–MS, and HRLC–MS analyses, including temperature programs, flow rates, and ion source settings, are summarized in Supplementary Information SI 2.

#### Determination of MIC, MBC, and MBIC

The minimum inhibitory concentration (MIC) and minimum bactericidal concentration (MBC) were determined using a microdilution assay in 48-well plates. Pathogens were treated with escalating doses of PF10, with 1% (2 × 10^3^ CFU/mL) of overnight culture used as inoculum. The MIC was recorded as the lowest concentration showing no visible growth after incubation. To determine MBC, 10 µL of 5 mg/mL MTT [3-(4,5-dimethylthiazol-2-yl)-2,5-diphenyltetrazolium bromide] was added to each well (control, treated, and blank) and incubated for 4 h; the concentration at which no formazan crystal formation occurred was considered the MBC, indicating complete bacterial killing (Benov 2021, 2019; Grela et al. 2018). MTT assay was further complemented with agar streak method to conclude the MBC value.

The minimum biofilm inhibitory concentration (MBIC) was determined using a similar microdilution approach. After treatment and incubation, wells were washed three times with sterile distilled water to remove planktonic cells, air-dried, and stained with 0.4% crystal violet. After washing and drying, crystal violet bound to biofilms was solubilized with 20% (v/v) glacial acetic acid, and absorbance was measured at 570 nm. MBIC was defined as the lowest concentration showing ≥ 80% inhibition of biofilm biomass compared to untreated control.

For microscopic visualization, biofilms were grown on sterile glass slides placed in 24-well plates containing 1 mL of MH broth, with or without active fraction. After 24 h incubation at 37 °C, slides were washed with PBS, air-dried, stained with 0.4% crystal violet for 10 min, washed to remove excess stain, and imaged under a light microscope (Olympus CX43, Tokyo, Japan) at 400 × magnification using a digital camera (Magcam DC 10, New Delhi, India) (Cruz et al. 2018; She et al. 2015).

## Anticancer studies

### Determination of anti-proliferative effect

Anti-proliferative capacity was examined as per standard protocol employing 3-(4, 5-dimethylthazol-2yl)-2, 5-diphenyl tetrazolium-bromide (MTT).

#### Nuclear condensation assay

Hoechst 33,342 was used for determining nuclear condensation. Cancer cells were seeded at 10,000 cells/well in 24 well plates. Media was aspirated after incubation and treated with different concentrations of the active fraction. After 24 h, each well was incubated with Hoechst 33,342 (5 µg/mL) for 15 min at 37 °C in carbon dioxide incubator. The wells were washed twice in PBS and were analysed through fluorescence microscope (Nikon, Japan) under UV filter sets, image captured and analysed using NIS Elements Version 0.410.

#### Western blot

The antibodies for PARP (Cat-ID: 9542; Dilution: 1;1000), Bax (Cat-ID:2772; Dilution: 1;1000), Caspase 3 (Cat-ID:9662; Dilution: 1;1000), β-actin (Cat-ID:4967; Dilution: 1;1000), and Caspase 9 (Cat-ID:9508; Dilution: 1;1000) were purchased from Cell signalling technology, USA. Proteins were separated by SDS-PAGE (10–12%) and transferred to a PVDF membrane. The membrane was blocked with 5% skim milk in TBST for 1 h at room temperature, followed by overnight incubation at 4 °C with primary antibodies. After washing with TBST, the membrane was incubated with HRP-conjugated anti-mouse secondary antibody for 1 h at room temperature. Protein bands were developed using standard procedures.

#### Statistical analysis

Experiments were carried out in both biological and technical triplicates for statistical significance. Student’s t-test was employed to examine data with statistical significance set at *P* < 0.05. For comparison between control and treatment groups in anti-proliferation assay non-linear aggression (curve fit) was employed. Statistical analysis was carried out in Graph Pad Software Inc. (La Jolla, CA, USA) Version 9.0.0. The results are presented as mean ± standard error of the mean (SEM).

## Results and discussion

### Isolation of actinomycetes and preliminary screening

The study isolated 24 actinomycete-like isolates, among this the strain designated WPF1 emerged as the most potent, showing the highest antimicrobial activity and was therefore selected for detailed investigation. The other isolates, along with their preliminary screening results, are provided in Supplementary Information (SI 3 and SI 4); they were stored for future research. Overall, 11 (45%) isolates exhibited activity against Gram-positive bacteria, while only two isolates (8%) were effective against both Gram-positive and Gram-negative bacteria, with T1 and WPF1 demonstrating broad-spectrum activity.

**Fig. 1:**
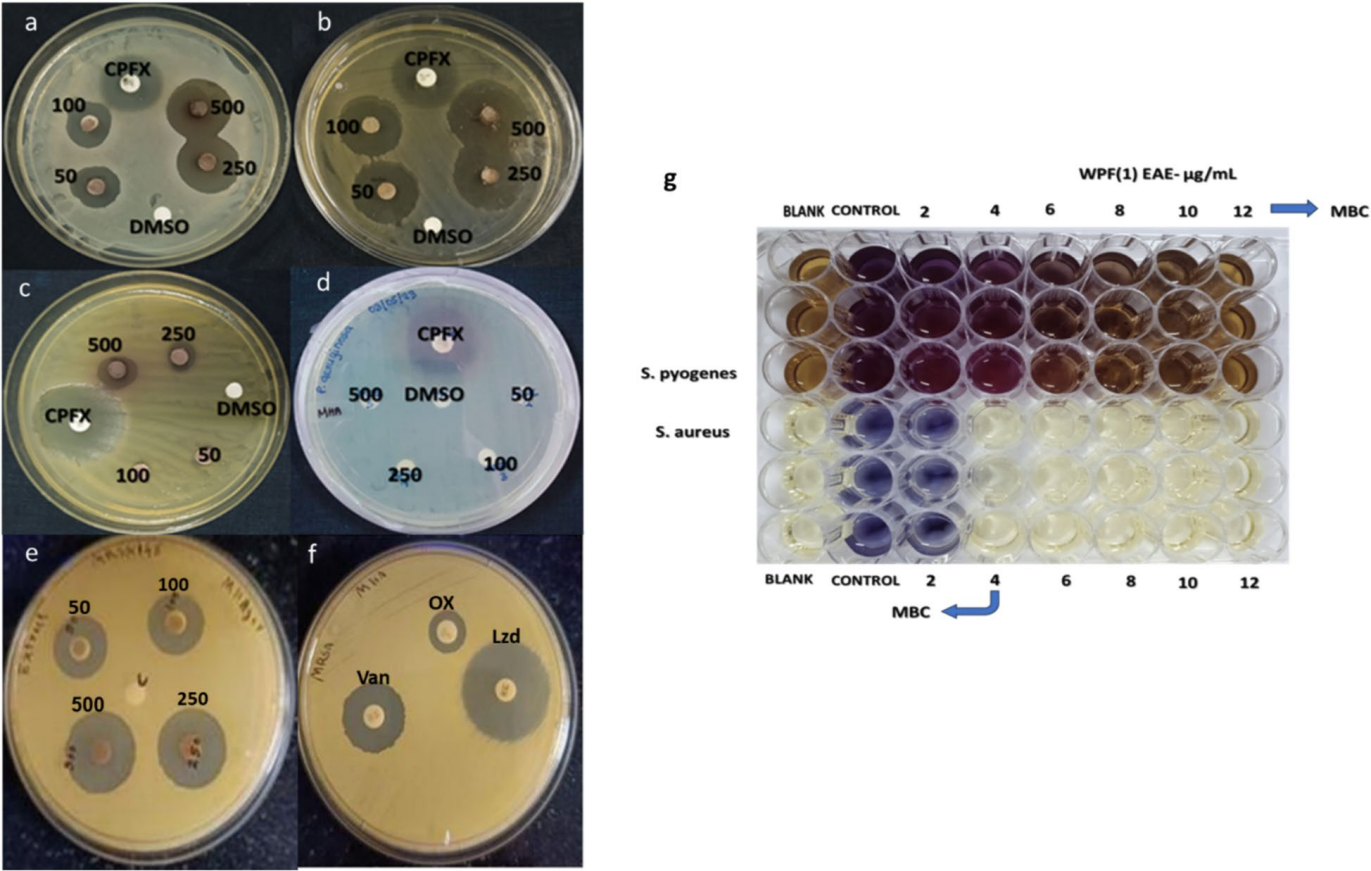
Antimicrobial activity of ethyl acetate extract of WPF1.Disc assay of crude extract against **a** *S. aureus*; **b** *S. pyogenes*; **c** P*.vulgaris*; **d** *P.aeruginosa*; **e** *S. aureus (MRSA S145)*; **f** Standard antibiotics against MRSA; **g** MBC determination via MTT assay of ethyl acetate extract of WPF1 against *S. aureus* and *S. pyogenes*. CPFX: Ciprofloxacin, DMSO: Dimethyl sulfoxide (negative control), Oxa: Oxacillin, Van: Vancomycin, Lzd: Linezolid

In disc diffusion assay, WPF1 exhibited the highest antimicrobial activity, with strong inhibition of *S. pyogenes* and *S. aureus* at 500 µg, moderate inhibition against some Gram-negative bacteria and fungi, and no significant activity against *P. aeruginosa, V. cholerae*, and Candida species. Importantly, WPF1 extract inhibited multidrug-resistant *S. aureus* (MRSA S145), producing a 21.17 ± 0.44 mm zone of inhibition at 500 µg, significantly surpassing the positive control, Oxacillin (6 mm).The extract showed a clear concentration-dependent effect, with MIC values of 10 µg/mL and 4 µg/mL for *S. aureus* and *S. pyogenes*, respectively, and MBC values similar to MIC. Positive controls (Ciprofloxacin and Nystatin) served as assay standards. (Table 1, Fig. 1g).

**Table 1:** Antimicrobial activity of the ethyl acetate extract of WPF1, measured as the zone of inhibition (in mm) against various organisms at concentrations of 50, 100, 250, and 500 µg

Human pathogens	Zone of inhibition in mm					
	Positive control	50 µg	100 µg	250 µg	500 µg	Negative control
*S.pyogenes*	21.33 ± 0.33	20.50 ± 0.29	21.50 ± 0.29	22.17 ± 0.17	30.17 ± 0.73	0
*S.aureus*	20.67 ± 0.33	15.17 ± 0.17	16.33 ± 0.33	20.17 ± 0.44	25.00 ± 0.29	0
*MRSA S145*	^a^Oxa-6.50 ± 0.28 ^b^Van-20 ± 0.58 ^c^Lzd-25.33 ± 0.88	16.50 ± 0.29	17.17 ± 0.17	20.17 ± 0.44	21.17 ± 0.44	0
*B.subilitis*	27.88 ± 0.17	5.17 ± 0.44	7.67 ± 0.33	11.17 ± 0.44	19.50 ± 0.58	0
*E-coli*	30 ± 0.58	0	0	9.25 ± 0.14	18 ± 0.29	0
*P.aeruginosa*	15.83 ± 0.44	0	0	0	0	0
*Serretia*	27.17 ± 0.60	0	0	0	0	0
*P.vulgaris*	27.33 ± 0.44	0	0	9.92 ± 0.22	11.75 ± 0.33	0
*E-feacalis*	24.17 ± 0.60	0	4.75 ± 0.25	9.42 ± 0.30	12.00 ± 0.58	0
*V.cholerae*	20.33 ± 0.33	0	0	0	0	0
*V. fluvialis*	21.17 ± 0.44	0	0	0	0	0
*C.albicans*	18.17 ± 0.17	0	0	0	0	0
*C.Kruzii*	18.50 ± 0.29	0	0	0	0	0
*A.niger*	18.83 ± 0.44	0	0	0	12.17 ± 0.44	0

^**a**^Oxa- Oxacillin

^**b**^vancomycin

^**c**^Lzd- Linezolid

WPF1 produced a distinct dark brown pigment on multiple media (ISP2, PDA, SCA, SMA, and NA) with varying intensities in the presence of CaCO_3_. Certain Streptomyces species produce melanoid pigments via conversion of tyrosine to DOPA-melanin, which is often linked to broad biological activities (Nirwati et al. 2022). Although pigment production has been associated with bioactivity in some Streptomyces spp.,(Nirwati et al. 2022), this observation in WPF1 requires further clarification and additional experimental studies.

### Whole genome analysis and phylogenetic assessment

**Fig. 2:**
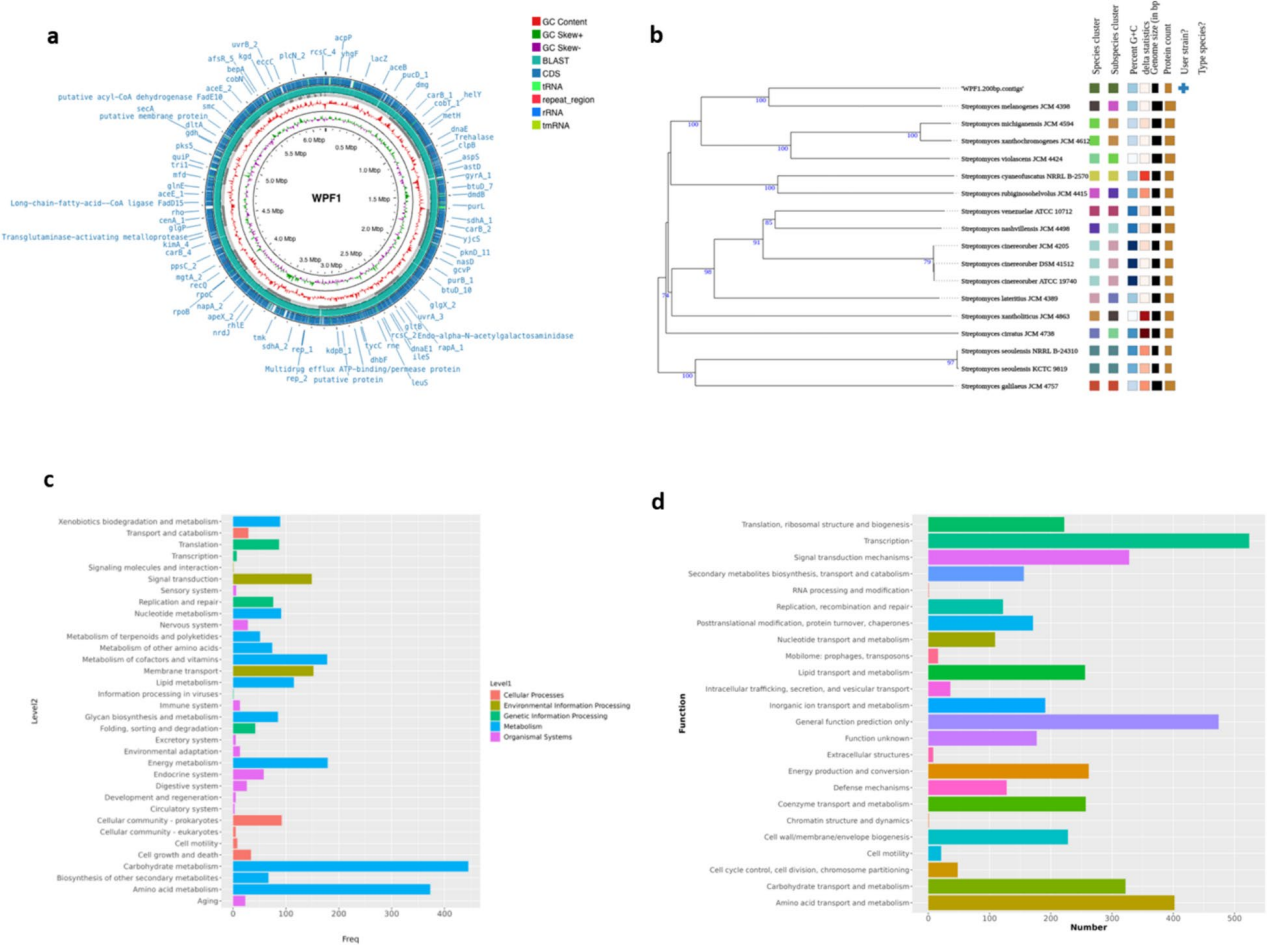
Whole genome analysis of WPF1. **a** Circular genome map, **b** Phylogenetic tree, **c** KEGG pathway classification, **d** COG functional categorization

Whole genome sequencing (WGS) of WPF1 yielded a high-quality assembly with a GC content of 71% and robust annotation revealing 5,395 coding sequences, 74 tRNAs, 4 rRNAs, and 29 miscellaneous RNAs (see Supplementary Information SI 5 for detailed metrics, including raw read counts and BUSCO analysis)**.** The circular genome map (Fig. 2a) illustrates annotated protein-coding genes such as putative acyl-CoA dehydrogenase, long-chain-fatty-acid-CoA ligase, and transglutaminase-activating metalloprotease. Inner rings represent GC content and GC skew, while color-coded tracks indicate CDS, tRNA, rRNA, tmRNA, and repeat regions. The genome achieved ~ 379-fold coverage, ensuring high reliability of the assembly.

The phylogenetic analysis of WPF1 revealed its close relation to *S. melanogenes* JCM 4398 and *S. michiganensis* JCM 4594, which are known for antibiotics production (Song et al. 2023; Held and Kutzner 1991). The whole genome-based phylogenetic analysis (Fig. 2b) indicates that WPF1 is grouped with high confidence values. The rMLST analysis confirmed the placement of WPF1 within the Actinomycetes with 100% support. Taxonomically, WPF1 is classified within the genus *Streptomyces***.** So based on whole genome sequencing (WGS) and phylogenetic tree analysis, the strain was designated as *Streptomyces melanogenes* WPF1.

Comparative insights highlight that closely related *S. melanogenes* strains from Henan Province, China (YBS22, yielding antimycin analogue) and Komba, Setagaya-Ku, Tokyo (n.sp., yielding melanomycin) have demonstrated antimicrobial efficacy primarily against plant pathogens (Song et al. 2023; Sugawara and Onuma 1957). In contrast, WPF1 shows potent activity against human pathogens, underscoring its novelty and potential for therapeutically relevant bioactive compounds.

### KEGG and COG annotation

The genome of *S. melanogenes* WPF1 was annotated using both the KEGG (Kyoto Encyclopedia of Genes and Genomes) and COG (Clusters of Orthologous Groups) databases, classifying the organism’s genes into various functional categories. These annotations provide insights into the organism’s metabolic capabilities, genetic information processing, and environmental adaptability.

In total, 2,611 genes were classified into 25 functional categories in KEGG, while 3,892 genes were categorized in COG (Fig. 2c). Metabolic pathways were most prominently represented in both analyses, with carbohydrate and amino acid metabolism being the most enriched, indicating the organism’s strong capacity for energy production and nutrient assimilation. Lipid metabolism, secondary metabolite biosynthesis (including terpenoid and polyketide pathways), and energy metabolism were also well represented, underscoring the potential of WPF1 for bioactive compound production.

Environmental information processing was dominated by membrane transport and signal transduction pathways, suggesting robust nutrient uptake and sophisticated responses to environmental cues (Fig. 2c, 2d). Cellular processes included genes for cell motility, growth, and death, supporting WPF1’s adaptability and potential for biofilm formation. Genetic information processing pathways were enriched for translation, transcription, and replication, reflecting active protein synthesis and genomic maintenance. KEGG also annotated genes for environmental adaptation and immune system function, suggesting stress tolerance and survival mechanisms.

A detailed breakdown of gene counts for each KEGG subcategory and COG functional group is provided in Supplementary Information SI 6.

### antiSMASH analysis of WPF1

antiSMASH (antibiotics and Secondary Metabolite Analysis Shell) is a comprehensive bioinformatics tool designed to identify and analyze biosynthetic gene clusters (BGCs) in microbial genomes. It predicts the potential of microorganisms to produce secondary metabolites, such as antibiotics, antifungals, and other bioactive compounds, by comparing genomic data to known BGCs. The tool also provides detailed annotations and visualizations, enabling researchers to explore the biosynthetic capabilities of microorganisms effectively (Blin et al. 2019).

The genome of *S.melanogenes* WPF1 harbors 26 biosynthetic gene clusters as identified by antiSMASH. Notably, the clusters with the most similarity index were an ectoine cluster (100% similarity) known for stress-protective functions (Li et al. 2024), a NI-siderophore cluster (100% similarity to desferrioxamin B) with potential antimicrobial applications (Bellotti and Remelli 2021), and several terpene clusters including one with 84% similarity to hopene (Kongue et al. 2013). Particularly significant is the Type II polyketide synthase (T2PKS) cluster showing 97% similarity to kinamycin (Smitka et al. 1992), a well-known antibiotic, highlighting the strain’s potential as a source of therapeutically relevant compounds. Another T2PKS cluster was identified to have 62% similarity with alnumycin group of antibiotics. The genome also contains multiple other low similarity PKS clusters (T1PKS, T3PKS, and PKS-like). The presence of NRPS and RiPP-like clusters, collectively suggesting a broad capacity for secondary metabolite biosynthesis. Clusters with low (< 20%) similarity to known compounds were considered as candidates warranting further characterization (Deepthi et al. 2023). A complete list of all 26 BGCs is provided in Supplementary Information SI 7.

### Bioactivity guided partial purification

**Fig. 3:**
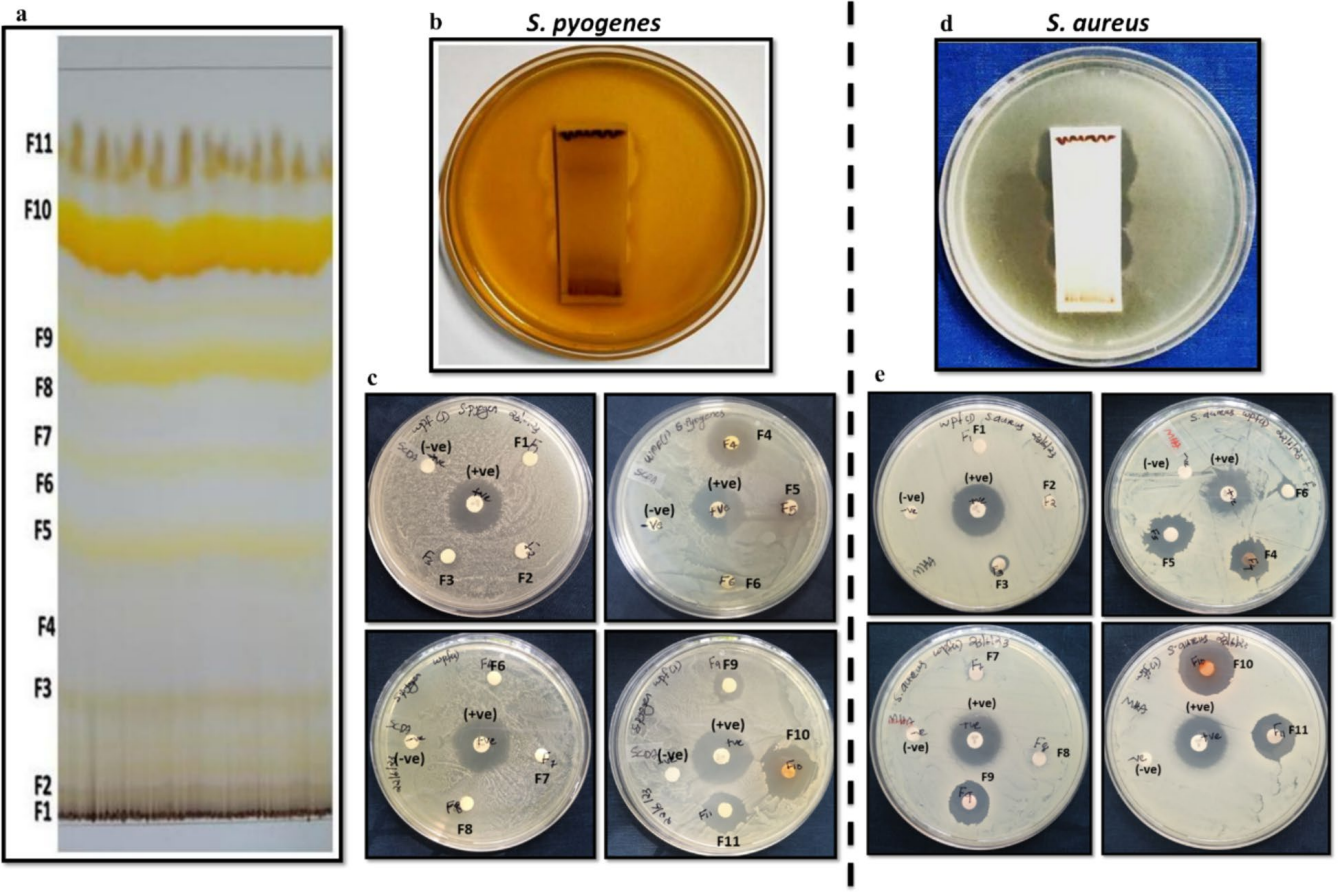
Bioactivity-guided partial purification of WPF1 extract. **a** TLC profile showing 11 fractions (F1–F11); **b**,**d** Bioautography against *S. pyogenes* and *S. aureus*; **c**,**e** Zone of inhibition of fractions; F10 showed highest activity and was selected for further purification. Controls: DMSO (-), positive ( +)

Bioautography technique was employed to identify antimicrobial fractions on TLC. The mobile phase of chloroform:acetone (7:3) provided the best separation of the extract into distinct fractions (Fig. 3a), and the Rf values of each separated fraction were recorded and are presented in Table 2. Fractions were designated as F1–F10 based on reproducibility of the specific TLC bands over time. Several fractions (500 μg/disc) showed clear zones of inhibition against both *S. pyogenes* and *S. aureus* in TLC-Direct Bioautography (Fig. 3b, d), and the fractions detected with antimicrobial activity were further scrutinized. Seven fractions—F3, F4, F5, F6, F9, F10, and F11—exhibited evident activity in the disc diffusion assay at a concentration of 500 μg/disc. Among these, fraction F10 demonstrated the highest zone of inhibition, measuring 22 ± 0.29 mm against *S. aureus* and 20 ± 0.29 mm against *S. pyogenes*. Overall, the seven active fractions produced a zone of inhibition of 10 mm or more against one or both pathogens (Table 2 & Fig. 3c, e).

**Table 2:** Zone of inhibition of TLC fractions against *S. pyogenes* and *S aureus*

TLC fractions (500 μg/disc)	Rf value	Zone of inhibition(mm)	
		*S.pyogenes*	*S.aureus*
F1	0.016	0	0
F2	0.044	0	0
F3	0.172	0	7
F4	0.3	17.83 ± 0.17	15.00 ± 0.29
F5	0.366	11 ± 0.29	16.17 ± 0.44
F6	0.438	0	7.17 ± 0.17
F7	0.477	0	0
F8	0.566	0	0
F9	0.627	13 ± 0.29	15 ± 0.58
F10^*^	0.8	20 ± 0.29	22 ± 0.29
F11	0.911	12.50 ± 0.29	16.42 ± 0.30
Positive control		21.33 ± 0.17	20.50 ± 0.29
Negative control		0	0

^*^F10 showed the highest inhibition zone against both pathogens

Fraction F10 was further taken up for purification of the therapeutic molecule. The HPLC-purified product of F10, designated as Partially purified Fraction F10 (PF10), eluted at 23.716 min, was collected, lyophilized, and subsequently subjected to GC–MS and LC–MS analyses for compound identification and bioactivity evaluation. PF10 collected by Preparative HPLC exhibited the strongest antimicrobial activity. The PF10 was employed for further biological studies.

### Metabolomic profiling employing GC–MS, LC–MS and HRLCMS-QToF

The GC–MS analysis of PF10 revealed a diverse profile of lipid-derived compounds (Table 3), with Oleic acid being the most prominent, constituting 53.18% of the total volatile compounds detected in the GC–MS chromatogram.Oleic acid is a monounsaturated fatty acid known for its potential antimicrobial activity, primarily by disrupting microbial cell membranes (Ramadan et al. 2024). Oleic acid and its derivatives have also been reported from biological extracts that can contribute to antimicrobial activity against human pathogens (Gama et al. 2025). The other bioactive compound identified was 1-cis-Vaccenoylglycerol, which belongs to the monoacylglycerol group; this class of compounds has also been previously reported from Streptomyces. Microbes, including Streptomyces species, harbor biosynthetic pathways that produce precursors for monoacylglycerols like fatty acyl-CoA and other long-chain unsaturated molecules (Cunha et al. 2024). Monoacylglycerols are more effective on Gram-positive pathogens and can inhibit bacterial growth via membrane disruption and interference with signal transduction (Preuss et al. 2005; Tesser et al. 2020). Thus, the GC–MS profile suggests the presence of lipids that could contribute to membrane-disruptive effects.

**Table 3:** Compounds identified in Fraction PF10 of the ethyl acetate extract of *S. melanogenes* WPF1, analysed by GC–MS

RetentionTime(min)	Area (%)	Height (%)	Compound name
32.655	53.18	21.43	Oleic acid
37.538	1.71	3.3	1-cis-Vaccenoylglycerol

Orbitrap LC–MS analysis of the bioactive PF10 fraction identified Kinamycin D, L-α-Palmitic acid, and Oleic acid (Table 4). Kinamycin D is a well-known antibiotic and anticancer molecule. AntiSMASH analysis further confirmed the presence of a kinamycin biosynthetic gene cluster in *S. melanogenes* WPF1, supporting its biosynthetic origin. The lipidic compounds L-α-Palmitic acid (hexadecanoic acid) and Oleic acid were also detected as co-occurring metabolites. These molecules are known to disrupt bacterial membranes and interfere with biofilm formation by incorporating into lipid bilayers, thereby compromising membrane structure and integrity (Kumar et al. 2020; Yoon et al. 2018; McGaw et al. 2002).

**Table 4:** Compounds identified in Fraction PF10 of the ethyl acetate extract of *S. melanogenes* WPF1, analysed by Orbitrap LC–MS

Compound	Mol. formula	RT	MW	Area
Kinamycin D	C_22_H_18_N_2_O_9_	0.89	454.102	1.1E + 10
L-α-Palmitic acid	C_19_H_38_O_4_	0.7	330.277	9E + 09
Oleic acid	C_18_H_34_O_2_	1.01	282.256	2E + 07

HRLC–MS analysis of the PF10 fraction identified bioactive compounds like Kinamycin D and Palmitic acid each with potential therapeutic relevance (Table 5). Kinamycin D was confirmed based on accurate mass, molecular formula and MS/MS fragmentation (see Supplementary Information SI 8). Palmitic acid, a fatty acid with reported antimicrobial and antibiofilm activity, was also detected in Orbitrap data. Palmitic acid was earlier reported in high amounts in *Streptomyces* sp. GMR22 via LC-HRMS, thus providing antibiofilm and antimicrobial activity to the chloroform extract obtained from the culture broth(Nirwati et al. 2022).

**Table 5:** Compounds identified in Fraction PF10 of the ethyl acetate extract of *Streptomyces melanogenes* WPF1, analyzed by HRLC-MS Q-ToF

Compound name	Formula	Calculated MW	Abundance
Kinamycin D	C_22_ H_18_ N_2_ O_9_	454.1036	381,412
Palmitic acid	C_16_ H_32_ O_2_	256.2412	......

Kinamycin D, a well-known antitumor antibiotic, was confirmed as one of the most abundantly detected metabolite through both Orbitrap LC–MS and HRLC–MS QToF analyses (Table 4 and Table 5), and its biosynthetic origin was supported by the presence of a Type II polyketide synthase (T2PKS) gene cluster identified by antiSMASH. Additionally, the partially purified fraction PF10 was separated by TLC using a chloroform: ethyl acetate (3:2) solvent system, where the resulting yellowish-orange band exhibited an Rf value of 0.39 (Hata et al. 1971), identical to that of Kinamycin D, further corroborating its presence in PF10. Overall, these complementary findings indicate that Kinamycin D is a contributor to PF10 bioactivity, with fatty acids as potential co-factors, that warrants further experimental and functional validation.

### Determination of MIC, MBC, and MBIC

**Fig. 4:**
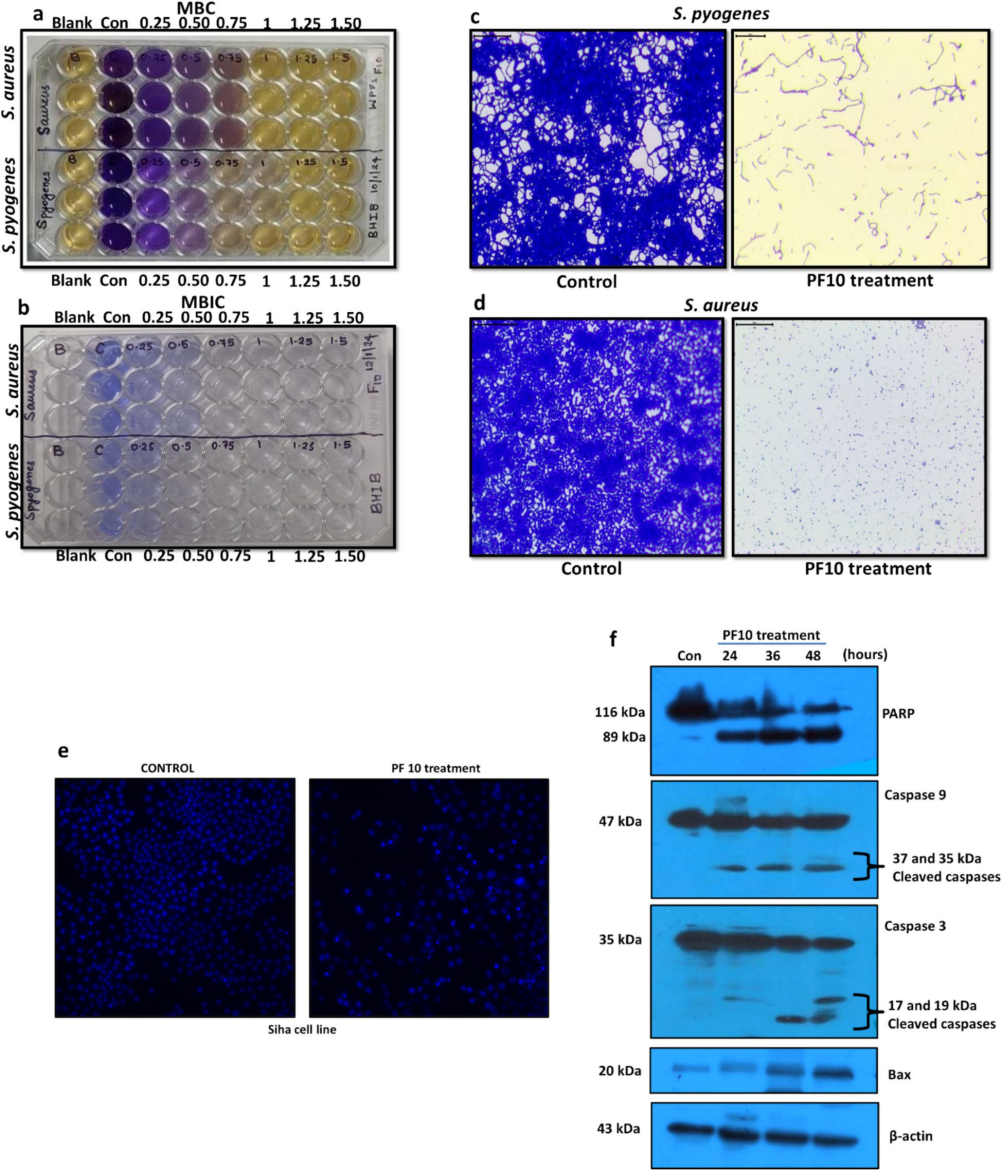
Antimicrobial, antibiofilm, and anticancer activities of PF10.a MTT assay for determining Mimimum Bactericidal Concentration (MBC) of *S. aureus* and *S. pyogenes*. **b** Crystal violet biofilm inhibition assay to analyze MBIC for *S. aureus* and *S. pyogenes*. **c**,** d** Microscopy of biofilm inhibition of *S. pyogenes* and *S. aureus*. Anticancer assessment. **e** Hoechst staining revealed nuclear condensation in Siha cells upon PF10 treatment. **f** western blot in SiHa cells showed induction of apoptosis via prominent markers (Full length blot images provided in Supplementary Information SI 9)

The minimum inhibitory concentration (MIC) and minimum bactericidal concentration (MBC) of the active fraction PF10 against *Staphylococcus aureus* were both determined to be 0.75 µg/mL. For *Streptococcus pyogenes*, the MIC was 0.75 µg/mL, while the MBC was slightly higher at 1 µg/mL (Fig. 4a). The antibiofilm efficacy of PF10 was also evaluated. At a concentration of 0.5 µg/mL, PF10 exhibited 89.3% inhibition of biofilm formation by *S. pyogenes*, whereas 93.6% inhibition was observed against *S. aureus* at 0.75 µg/mL (Fig. 4b). Microscopy studies of biofilm inhibition in both pathogens revealed that effective removal of biofilms were achieved in the respective MBIC values, thereby confirming antibiofilm attribute of PF10 (Fig. 4c, d). Complete inhibition of biofilm formation was achieved at a concentration of 1 µg/mL (Fig. 4b). These findings suggest that PF10 demonstrates greater efficacy against Gram-positive bacterial pathogens.

Previous studies have reported that various kinamycin derivatives—specifically kinamycins A, B, C, and D—isolated from *Streptomyces murayamaensis* display selective antimicrobial activity against Gram-positive microorganisms (Omura et al. 1973). This observation aligns with the genomic and metabolic profiling data of WPF1, suggesting that a kinamycin-like compound may contribute to the antimicrobial activity observed with PF10. Omura et al. (1972) reported that Kinamycin D exhibited MIC values ranging from 0.12 to 0.24 µg/mL against several *S. aureus* strains (Omura et al. 1973). Notably, the antibiofilm activity of kinamycins remains largely unexplored, and the present study highlights their potential as promising candidates for further therapeutic development. It can also be noted here that scientific literature suggests that Kinamycins were never explored for its efficiency against *Streptococcus pyogenes*. Fatty acids like Oleic acid and palmitic acid have selective activity for gram-positive pathogens (Dilika et al. 2000; Desbois and Smith 2010; Khan et al. 2023**),** thus validating the results observed in our study.

### Evaluating anticancer efficiency

Treatment with PF10 resulted in half-maximal inhibitory concentrations (IC_50_) of 2.57 ± 0.564 µg/mL for the DLD1 cell line and 2.07 ± 0.384 µg/mL for the Siha cell line. The active fraction was tested in normal cell line HEK293 resulting in an IC_50_ of 5.582 ± 1.842 µg/mL, thus confirming that PF10 is more sensitive towards cancer cell lines. Morphological changes indicative of cytotoxicity, such as cellular rounding, were observed under phase-contrast microscopy within 24 h of treatment. Given that the Siha cell line exhibited the lowest IC_50_ value (Dose response curve- Supplementary Information SI 10), it was selected for subsequent analyses to elucidate the involvement of apoptosis-associated proteins. To further investigate nuclear alterations, Hoechst staining assay was performed, revealing chromatin condensation and the presence of apoptotic bodies with fragmented nuclei in both cell lines, compared to their respective untreated controls (Fig. 4e). Western blot analysis demonstrated PARP cleavage, activation of caspase 3 and caspase 9 and Bax accumulation thus confirming apoptosis as the primary mode of cell death upon PF10 exposure (Fig. 4e). These findings suggest that PF10 contains a potent anticancer compound. Supporting data from whole-genome sequencing and liquid chromatography–mass spectrometry (LC–MS) identified Kinamycin as a constituent of the PF10 fraction. Previous studies have shown that Kinamycin A and C induce apoptosis in Chinese hamster ovary cells, an effect attributed to the presence of a reactive diazo group, which is believed to contribute to their cytotoxic properties (Hasinoff et al. 2006). Kinamycin F induced apoptosis in human osteosarcoma cell lines through caspase activation, cyclin D3 suppression and cell cycle modulation, while study by O’Hara (2007) explored reductive attributes of diazo group present in Kinamycin F that enabled cancer cell growth inhibition via DNA and protein damage (Bavelloni et al. 2017; O'Hara et al. 2007). The molecular mechanisms underlying the anticancer activity of these kinamycins have been extensively studied. Notably, no prior research has investigated the cytotoxicity of Kinamycin D or other kinamycins against colorectal and cervical cancer cell lines. These findings emphasize the need for further studies to elucidate its precise molecular mechanisms and expand its potential applications in cancer therapy. The fatty acids detected in the fraction were reported in many research studies for their anticancer property. Oleic was able to induce various antagonistic pathways depending on the cancer cell type leading to cell death (Giulitti et al. 2021). Oleic acid can cause mitochondrial dysfunction, autophagy, intrinsic and extrinsic mode of apoptotic pathways in cancer cells (Deng et al. 2023). Palmitic acid compromised membrane integrity of colon cancer cells leading to proliferation suppression and eventually induction of apoptosis via the mitochondrial (intrinsic) pathway (Wang et al. 2023).

## Conclusion

The isolate *Streptomyces melanogenes* WPF1, obtained from the Vagamon Pine Forest in the Western Ghats, exhibited significant antimicrobial activity, particularly against Gram-positive pathogens including *Staphylococcus aureus* and *Streptococcus pyogenes*. The partially purified metabolite fraction also demonstrated anticancer activity in colorectal and cervical cancer cell lines. Chromatographic separation coupled with high-resolution spectrometric analyses identified Kinamycin D as one of the bioactive metabolites. Kinamycins were first described in the 1970s from *S. murayamaensis*, and current evidence indicates that *S. melanogenes* WPF1 constitutes only the third reported natural source of this class of compounds. In addition, the fraction contained fatty acids such as oleic acid and palmitic acid, that has been earlier reported for antimicrobial and anticancer activity (Dilika et al. 2000; Desbois and Smith 2010; Khan et al. 2023; Deng et al. 2023; Wang et al. 2023). The present work is constrained by its dependence on partially purified fractions, the absence of mechanistic characterization, and the lack of in vivo validation. Future investigations should therefore focus on complete purification, mechanism-of-action studies, and animal model testing to establish the therapeutic relevance of Kinamycin D and other predicted metabolites. Overall, the bioactivities observed are attributable to a synergistic interaction of multiple metabolites within the active fraction. Collectively, these findings highlight *S. melanogenes* WPF1 as a promising microbial resource and emphasize the value of bioprospecting unique ecological niches such as the Vagamon Pine Forest of the Western Ghats for the discovery of novel antimicrobial and anticancer agents.

## Supplementary Information

Supplementary file 1

## Data Availability

The Whole Genome Sequence of *Streptomyces melanogenes* WPF1 has been deposited, the data are publically available at NCBI genbank under the BioProject accession number PRJNA1079905, and Bio sample accession number SAMN40094909 with the link https://www.ncbi.nlm.nih.gov/sra/PRJNA1079905

## Abbreviations

ATCC: American type culture collection
BGCs: Biosynthetic gene clusters
BLAST: Basic local alignment search tool
BUSCO: Benchmarking universal single-copy orthologs
CDS: Coding sequences
COG: Clusters of orthologous groups
DMSO: Dimethyl sulfoxide
GC: Guanine-cytosine
GC–MS: Liquid chromatography–mass spectrometry
HRMS: High-resolution mass spectrometry
HRP: Horseradish peroxidase
IC50: Half maximal inhibitory concentration
ISP2: International streptomyces production media 2
KEGG: Kyoto encyclopedia of genes and genomes
LC–MS: Liquid chromatography–mass spectrometry
MBC: Minimum bactericidal concentration
MBIC: Minimum biofilm inhibiting concentration
MHA/MHB: Mueller Hinton Agar/ Mueller Hinton Broth
MIC: Minimum inhibitory concentration
mRNA: Messenger RNA
MRSA: Methicillin resistant staphylococcus aureus
MTCC: Microbial type culture collection
MTT: 3-(4, 5-dimethylthazol-2yl)-2, 5-diphenyl tetrazolium-bromide
NRPS: Nonribosomal peptide-synthetase
OD: Optical density
PARP: Poly (ADP-ribose) polymerase
PDA/PDB: Potato Detrose Agar/Potato Dexrose Broth
PF10: Purified fraction 10
PVDF: Polyvinylidene difluoride
QUAST: Quality assessment tool
Rf: Retardation factor
rMLST: Ribosomal multilocus sequence typing
RP: Reverse phase
rRNA: Ribosomal RNA
SCA: Starch casein agar
SDS-PAGE: Sodium dodecyl sulphate–polyacrylamide gel electrophoresis
SI: Supplementary informations
T1/2/3PKS: Type I/II/III polyketide synthases
TLC: Thin layer chromatography
tmRNA: Transfer-messenger RNA
tRNA: Transfer RNA
YEME: Yeast extract mannittol media
