# Supplementary material for: Whole genome surveying and metabolomic profiling aided navigation of therapeutic plethora of Streptomyces melanogenes WPF1 isolated from Pinus patula rhizosphere
Source: Bioresour Bioprocess. 2025 Dec 10;12(1):149. doi: 10.1186/s40643-025-00973-7 (PMC12696212; doi:10.1186/s40643-025-00973-7)
Supplement: Supplementary file 1 [file 40643_2025_973_MOESM1_ESM.docx]

**SUPPLEMENTARY INFORMATION (SI)**

**Whole genome surveying and metabolomic profiling aided navigation of therapeutic plethora of *Streptomyces melanogenes* WPF1 isolated from *Pinus patula* rhizosphere.**

Abhirami ChithrakumariRanesan^a,bϕ^,Vipin Mohan Dan^a^*^ϕ^, Abhijeeth Muralidharan Nair^c^, Remya Pattaruparambil^a^, Athira Santhosh^a^, Sajith Raghunandanan^d^*, Sarika Ambika Rajendran^e^*

^a^Division of Microbiology, KSCSTE- Jawaharlal Nehru Tropical Botanic Garden and Research Institute, Pacha-Palode, Thiruvananthapuram 695562, Kerala, India

^b^Researchcenter, University of Kerala, Thiruvananthapuram - 695 014, Kerala, India

^c^Cancer Research Programme-4, Rajiv Gandhi Centre for Biotechnology (RGCB),
ThycaudPost, Poojappura,Thiruvananthapuram - 695 014, Kerala, India

^d^Department of public health and genomics, Manipal School of Life Sciences, Manipal Academy of Higher Education, Manipal, Karnataka - 576104, India

^e^Kerala Biotechnology Commission, KSCSTE, SasthraBhavan,Pattom, Thiruvananthapuram, Kerala 695004, India

**Correspondence:** Dr Vipin Mohan Dan- [vipindan@gmail.com](mailto:vipindan@gmail.com), [vipindan@jntbgri.res.in](mailto:vipindan@jntbgri.res.in) ;Dr Sajith Reghunandanan- [sajith.r@manipal.edu](mailto:sajith.r@manipal.edu) ;Dr Sarika Ambika Rajendran**-** [sarikaar.kscste@kerala.gov.in](mailto:sarikaar.kscste@kerala.gov.in)

^ϕ^Equal contribution

| **Pathogen** | **Strain No.** | **Type** | **Source** |
| --- | --- | --- | --- |
| *Streptococcus pyogenes* | ATCC 19615 | Gram-positive | ATCC |
| *Staphylococcus aureus* | MTCC 740 | Gram-positive | MTCC |
| *Bacillus subtilis* | MTCC 44 | Gram-positive | MTCC |
| *Enterococcus faecalis* | MTCC 439 | Gram-positive | MTCC |
| *Escherichia coli* | MTCC 443 | Gram-negative | MTCC |
| *Pseudomonas aeruginosa* | MTCC 741 | Gram-negative | MTCC |
| *Serratia marcescens* | ATCC 8100 | Gram-negative | ATCC |
| *Proteus vulgaris* | ATCC 6380 | Gram-negative | ATCC |
| *Vibrio cholerae* | MTCC 3904 | Gram-negative | MTCC |
| *Vibrio fluvialis* | MTCC 4432 | Gram-negative | MTCC |
| *Candida albicans* | MTCC 227 | Fungal yeast | MTCC |
| *Candida krusei* | ATCC 6528 | Fungal yeast | ATCC |
| *Aspergillus niger* | MTCC 1344 | Filamentous fungus | MTCC |

**SI 1**: Microbial strains used for antimicrobial and antifungal assays

**SI 2:** Detailed Protocol for Metabololmic profiling

**SI 2.1 GCMS Analysis**

Gas chromatography-mass spectrometry (GC-MS) analysis of PF10 was performed using a Shimadzu Nexis GC-2030 system equipped with an AOC-30/20i auto-sampler. The SH-I-5Sil MS column (30.0 m length, 0.25 mm inner diameter, 0.25 μm film thickness) was used for the chromatographic separation. The injection volume was 1.0 μL, and the injection mode was split with a split ratio of 20:1. The injector temperature was set at 260°C, and the oven temperature was programmed to increase from 80°C (4 min hold) to 280°C at a rate of 5°C/min, with a final hold of 6 minutes.

The carrier gas flow was controlled in linear velocity mode, with a column flow of 1.00 mL/min and a total flow of 23.9 mL/min. The ion source temperature of the mass spectrometer (MS) was maintained at 220°C, with an interface temperature of 280°C. The mass range was scanned from 35 to 500 m/z, using a scan speed of 1666. Compound identification was performed using the NIST 20 library, and data acquisition and analysis were managed using GCMS Solutions software.

**SI 2.2 LC-MS Analysis**

The LC-MS analysis of PF10 was carried out using a Vanquish UHPLC system coupled with an Eclipse Orbitrap mass spectrometer. A Waters RP column (2.1 x 150 mm, 1.8 µm particle size) was employed for chromatographic separation, with mobile phase A consisting of 0.1% formic acid in water and mobile phase B consisting of 0.1% formic acid in methanol. The column temperature was maintained at 45°C, and the auto sampler temperature was set at 5°C. The injection volume was 5 µL, with a total run time of 15 minutes. The flow rate was 0.350 mL/min, and a gradient method was used with mobile phase B starting at 0.5% and increasing to 98% over 6 minutes, held for 7 minutes, and then returning to 0.5%.

The mass spectrometer was equipped with a heated electrospray ionization (H-ESI) source. The ion source operated with a spray voltage of 3400 V in positive mode and 2800 V in negative mode. The sheath gas flow was set to 40 Arb, and the auxiliary gas flow was set to 5 Arb, with the ion transfer tube temperature maintained at 300°C and the vaporizer temperature at 400°C. Data acquisition was performed in both positive and negative ion modes, scanning a mass-to-charge ratio (m/z) range of 100–1000. The resolution was set at 120,000, and MS/MS fragmentation was achieved using higher-energy collisional dissociation (HCD) with collision energy of 35%. Data processing was performed using Compound Discoverer 3.3 software, which identified and quantified features across all samples. Compound identification was aided by databases such as mzCloud, ChemSpider, KEGG, and LIPID MAPS.

**SI 2.3 HRLC-MS-QTOF**

High-resolution LC-MS analysis was performed on an Agilent QTOF system (USA) with a Hypersil GOLD C18 column (100 × 2.1 mm, 3 µm), with an MS Absorbance threshold of 200 and MS/MS Absorbance threshold of 5. Data were acquired and processed using Agilent Mass Hunter software (B.06). The mobile phases were 0.1% formic acid in Milli-Q water (A) and acetonitrile (B) at 0.3mL/min. The instrument operated in positive ion mode with a Dual AJS ESI source under standard parameters, and data were collected in AutoMS² mode over m/z 120–1200.

**Summary of GC–MS, LC–MS, and HRLC–MS Operating Condition**

| **Parameter** | **GC–MS** | **LC–MS** | **HRLC–MS–QTOF** |
| --- | --- | --- | --- |
|  |  |  |  |
| **Instrument** | Shimadzu Nexis GC-2030 | Vanquish UHPLC + Orbitrap Eclipse MS | Agilent QTOF (USA) |
| **Column** | SH-I-5Sil MS (30 m × 0.25 mm × 0.25 µm) | Waters RP (2.1 × 150 mm, 1.8 µm) | Hypersil GOLD C18 (100 × 2.1 mm, 3 µm) |
|  |  |  |  |
| **Sample Injection** | 1 µL, split mode (20:1) | 5 µL | – |
| **Column / Injector Temp** | Injector: 260 °C; Oven: 80 °C (4 min hold) → ramp 5 °C/min to 280 °C → hold 6 min | Column: 45 °C; Autosampler: 5 °C | – |
| **Mobile Phases** | – | A: 0.1% FA in water; B: 0.1% FA in methanol | A: 0.1% FA in Milli-Q water; B: acetonitrile |
|  |  |  |  |
| **Flow Rate** | 1.00 mL/min (column), 23.9 mL/min (total) | 0.350 mL/min | 0.3 mL/min |
| **MS Ionization / Source** | EI (Electron Ionization) | H-ESI (positive & negative) | Dual AJS ESI (positive) |
| **MS / Interface Temp** | Source: 220 °C; Interface: 280 °C | Ion transfer tube: 300 °C; Vaporizer: 400 °C | – |
| **Mass Range** | 35–500 m/z | 100–1000 m/z | 120–1200 m/z |
| **Resolution** | – | 120,000 | High-resolution |
|  |  |  | (AutoMS²) |
| **Other MS Settings** | Scan speed: 1666 | Sheath gas: 40 Arb; Aux gas: 5 Arb; Spray voltage: +3400 V / –2800 V; HCD CE: 35% | MS threshold: 200; MS/MS threshold: 5 |
| **Data Analysis** | GCMS Solutions software + NIST 20 library | Compound Discoverer 3.3; mzCloud, ChemSpider, KEGG, LIPID MAPS | Agilent MassHunter B.06 |


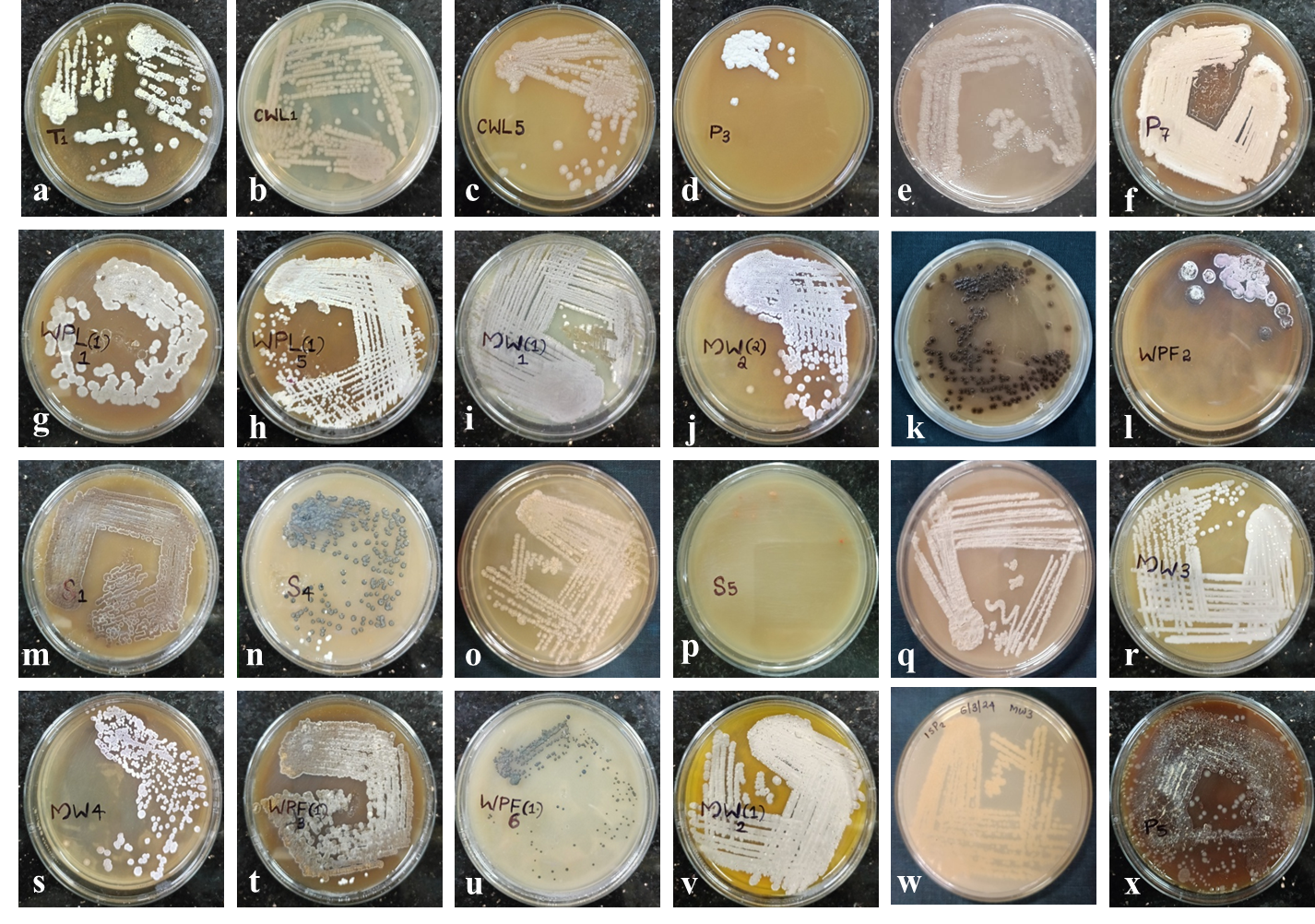


**SI 3:** Colony morphology of 24 different Actinomycetes isolates grown on ISP2 media. The figure illustrates the diversity in colony characteristics, including color, texture, and size of isolated actinomycete strains.(a) T1; (b) CWL1; (c) CWL5;(d) P3; (e) PLKNM1; (f) P7;(g) WPL1-1;(h) WPL1-5;(i) MW1-1;(j) MW2-2;(k) WPF1;(l) WPF2;(m) S1;(n) S4;(o) PLKNM2;(p) S5;(q) PLKNM4;(r) S6;(s) MW4;(t) WPF1-3;(u) WPF1-6;(v) MW1-2;(w) MW3;(x) P5.

.

| **Isolates** | ***S. pyogenes* ATCC700294D-5** | ***S. aureus* MTCC740** | ***E. coli* MTCC443** | ***P. aeruginosa* MTCC741** |
| --- | --- | --- | --- | --- |
| T1 | 12 | 18 | 10 | 7 |
| MW1-1 | 0 | 0 | 0 | 0 |
| MW1-2 | 0 | 0 | 0 | 0 |
| MW2-2 | 19 | 14 | 0 | 0 |
| MW3 | 16 | 11 | 0 | 0 |
| MW4 | 13 | 0 | 0 | 0 |
| CWL1 | 0 | 0 | 0 | 0 |
| CWL5 | 0 | 0 | 0 | 0 |
| PLKNM1 | 0 | 0 | 0 | 0 |
| PLKNM2 | 25 | 19 | 0 | 0 |
| PLKNM4 | 0 | 0 | 0 | 0 |
| WPL1-1 | 11 | 0 | 0 | 0 |
| WPL1-5 | 0 | 0 | 0 | 0 |
| WPF1 | 30 | 25 | 19 | 0 |
| WPF2 | 0 | 0 | 0 | 0 |
| WPF1-3 | 0 | 0 | 0 | 0 |
| WPF1-6 | 6 | 0 | 0 | 0 |
| P3 | 18 | 20 | 0 | 0 |
| P5 | 0 | 0 | 0 | 0 |
| P7 | 0 | 0 | 0 | 0 |
| S1 | 0 | 0 | 0 | 0 |
| S4 | 0 | 0 | 0 | 0 |
| S5 | 12 | 7 | 0 | 0 |
| S6 | 18 | 8 | 0 | 0 |

**SI 4:** Preliminary antibiotic screening of Actinomycete isolates from different sampling sites at varying altitudes. The table displays the inhibition zones (in mm) produced by each isolate against *Streptococcus pyogenes* ATCC 700294 D-5, *Staphylococcus aureus* MTCC 740, *Escherichia coli* MTCC 443, and *Pseudomonas aeruginosa* MTCC 741


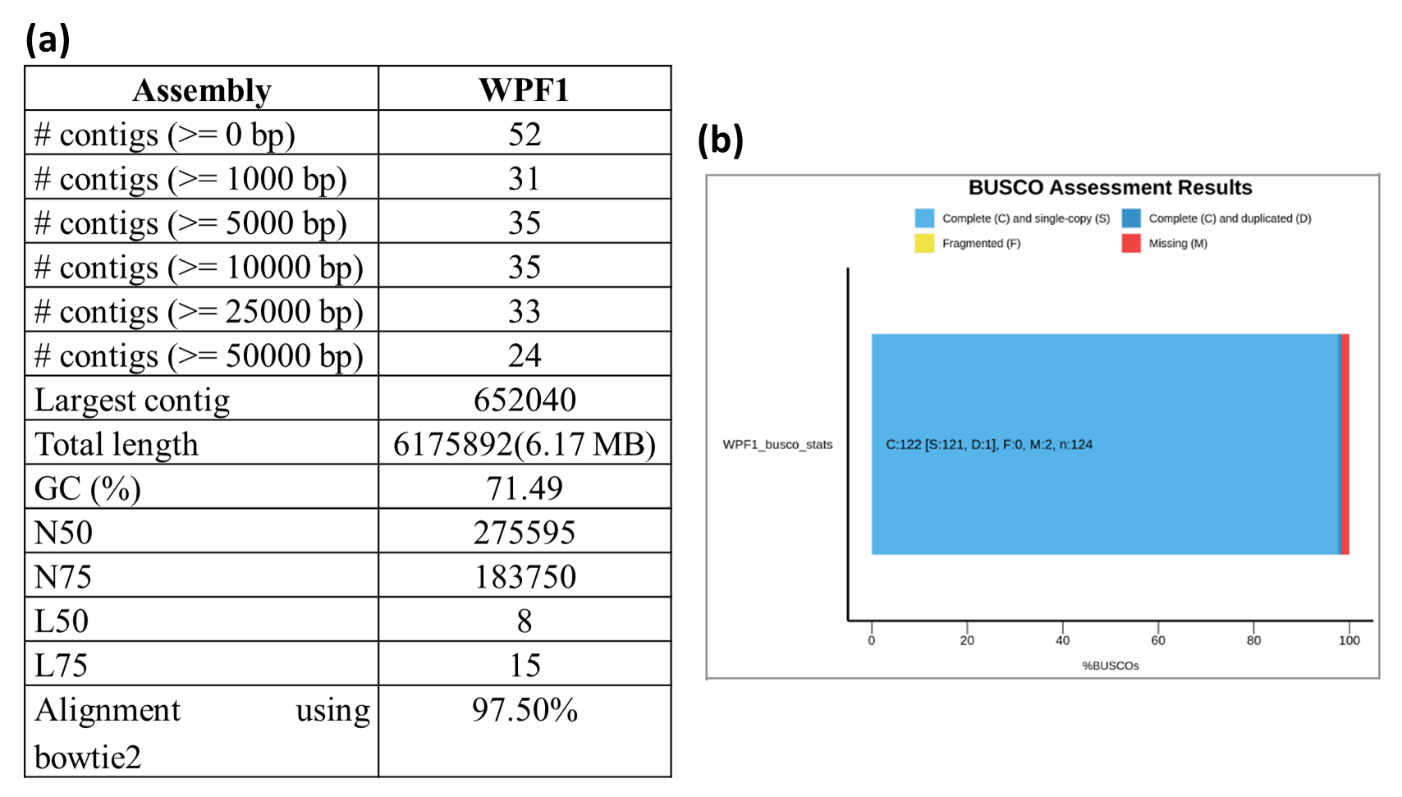


**SI 5:** Genome assembly statistics for WPF1**. (a)** The assembly consists of 52 contigs, with 31 contigs of at least 1000 bp and 24 contigs larger than 50,000 bp. The largest contig is 652,040 bp, contributing to a total genome length of 6.17 MB with a GC content of 71.49%. The N50 and N75 values are 275,595 bp and 183,750 bp, respectively, with corresponding L50 and L75 values of 8 and 15. Alignment using Bowtie2 revealed a 97.50% match to the reference sequences. **(b)** Summary of BUSCO anlaysis: The genome completeness was checked using BUSCO v5.3.2 . BUSCO provides a quantitative assessment of the completeness of expected gene content of a genome assembly.

| **Category** | **Subcategory** | **KEGG Gene Count** | **COG Gene Count** |
| --- | --- | --- | --- |
| **Metabolism** | Carbohydrate Metabolism | 423 | 262 |
|  | Amino Acid Metabolism | 346 | 224 |
|  | Lipid Metabolism | 182 | – |
|  | Secondary Metabolite Biosynthesis | 312 | 156 |
|  | Terpenoid & Polyketide Metabolism | 167 | – |
| **Environmental Information Processing** | Membrane Transport | 291 | 154 |
|  | Signal Transduction | 249 | 137 |
| **Cellular Processes** | Cell Growth & Death | 96 | – |
|  | Cell Motility | 117 | 87 |
| **Genetic Information Processing** | Translation | 133 | 95 |
|  | Transcription | 81 | 180 |
|  | Replication & Repair | 76 | 150 |
| **Organismal Systems** | Environmental Adaptation | 92 | 4 |
|  | Immune System | 47 | 3 |

**SI 6:** A detailed breakdown of gene counts for each KEGG subcategory and COG functional group

| **Region** | **Type** | **The most similar known cluster** | **Similarity** |
| --- | --- | --- | --- |
| Region 1.1 | Terpene | Hopene | 84.00% |
| Region 1.2 | T1PKS, hglE-KS | hexacosalactone A | 15.00% |
| Region 1.3 | T3PKS | accramycin A | 13.00% |
| Region 1.4 | PKS-like | toxoflavin/fervenulin | 14.00% |
| Region 3.1 | Terpene | TVA-YJ-2 | 16.00% |
| Region 3.2 | T3PKS | 7-deoxypactamycin | 22.00% |
| Region 4.1 | Butyrolactone | coelimycin P1 | 16.00% |
| Region 5.1 | RiPP-like | hexacosalactone A | 4.00% |
| Region 6.1 | Ectoine | Ectoine | 100.00% |
| Region 7.1 | NRPS | Q6402A | 20.00% |
| Region 7.2 | NI-siderophore | desferrioxamin B | 100.00% |
| Region 8.1 | CDPS | - | - |
| Region 11.1 | NI-siderophore, T2PKS | Kinamycin | 97.00% |
| Region 13.1 | NRPS | Murayaquinone | 31.00% |
| Region 13.2 | T2PKS | alnumycin A/alnumycin B/alnumycin C/alnumycin P/prealnumycin/thalnumycin A/thalnumycin B/K1115A/1,6-dihydro-8-propylanthraquinone | 62.00% |
| Region 14.1 | CDPS | BD-12 | 17.00% |
| Region 17.1 | T1PKS | Cyphomycin | 2.00% |
| Region 18.1 | NAPAA | ε-Poly-L-lysine | 100.00% |
| Region 20.1 | lanthipeptide-class-iii | SRO15-2212 | 57.00% |
| Region 22.1 | Melanin | 4-hydroxy-3-nitrosobenzamide | 57.00% |
| Region 26.1 | RiPP-like | - | - |

**SI 7:** antiSMASH analysis of biosynthetic gene clusters in Streptomyces melanogenes WPF1 and their similarity to known compounds


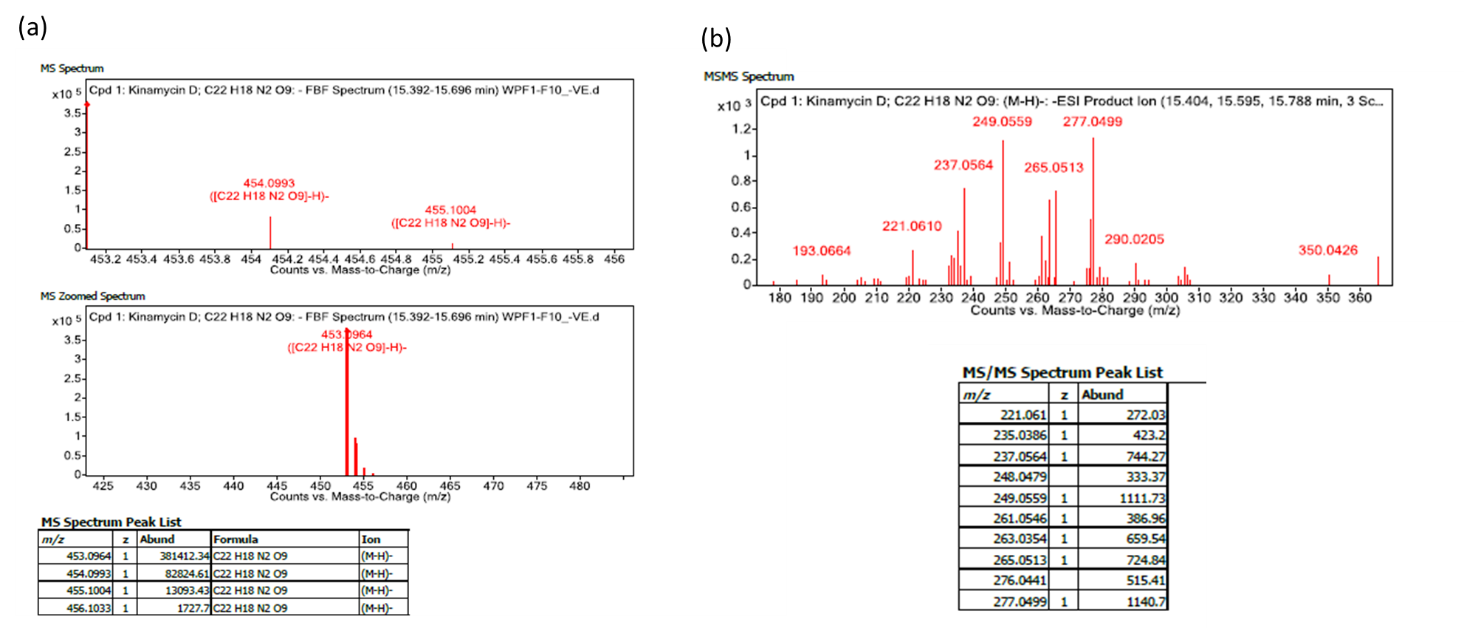


**SI 8**: (a) MS spectrum of Kinamycin D obtained from HRMS; (b) MS/MS spectrum- The HRMS fragmentation pattern of Kinamycin D


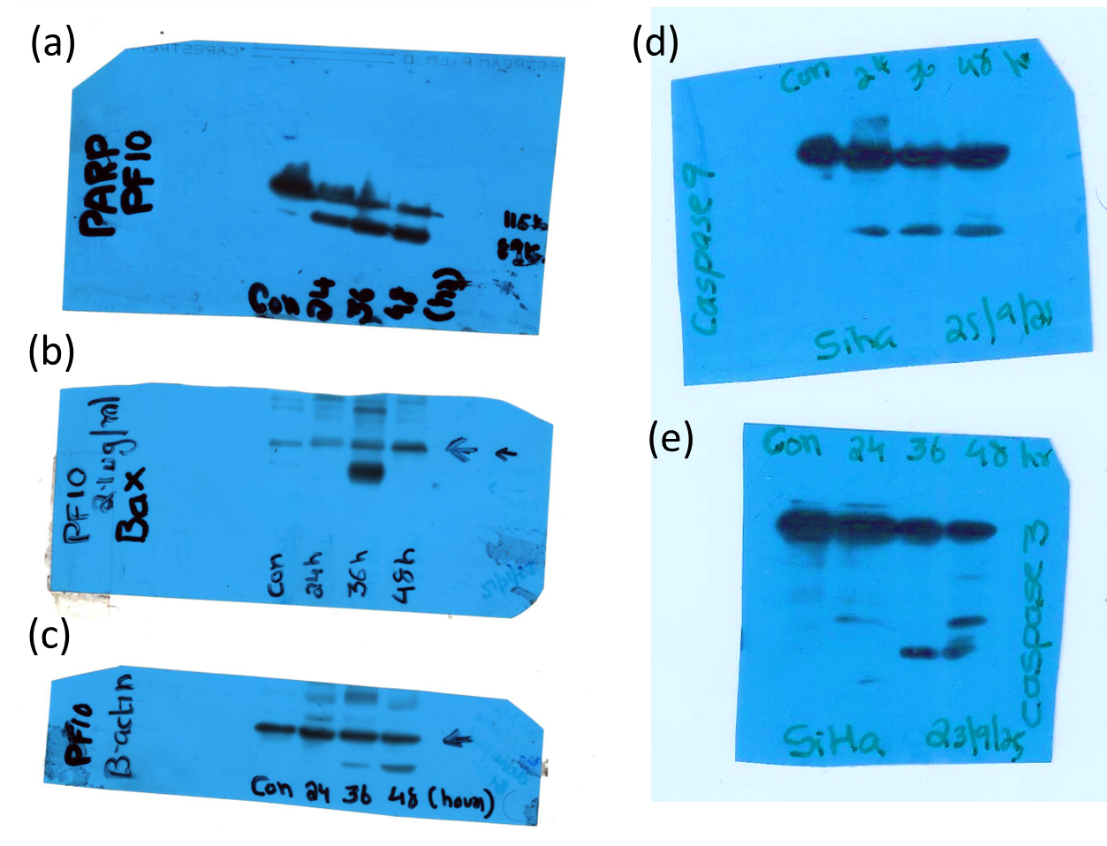


**SI 9:** Full length blots of apoptotic marker proteins:- PARP (a) ,Bax (b), control protein β- actin(c), (d) caspase 9 (d) and Caspase 3 (e).


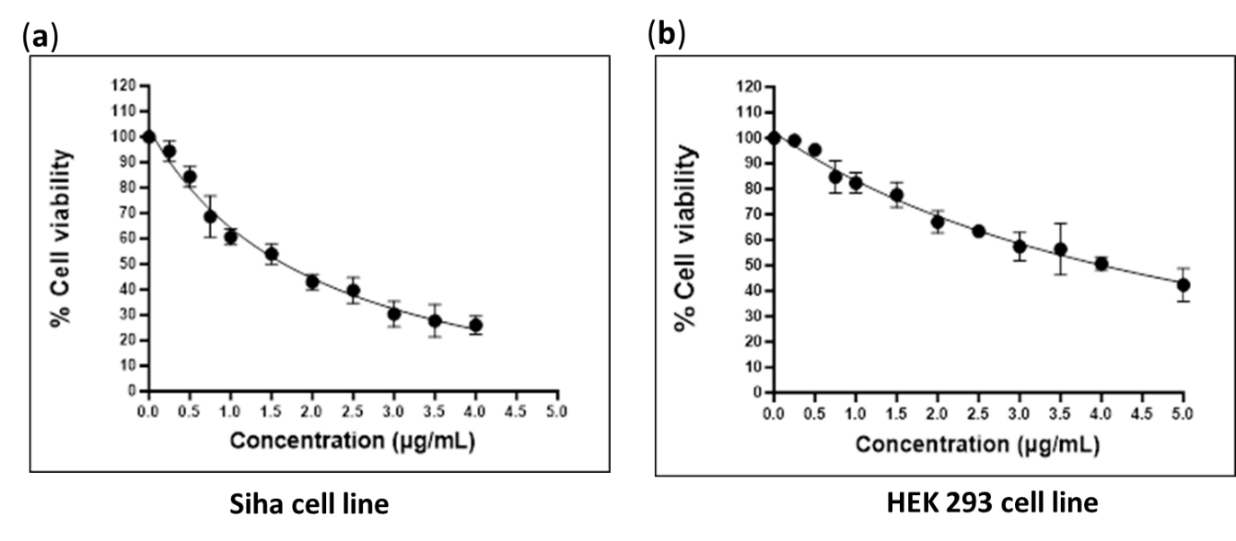


**SI 10**: Dose response curves- The active fraction PF10 exhibited an IC₅₀ value of 2.07 ± 0.384 µg/mL against the SiHa cancer cell line (a), while showing a higher IC₅₀ of 5.582 ± 1.842 µg/mL in the normal HEK 293 cell line (b), indicating greater selectivity and sensitivity toward the cancer cells
